# Explosive Spalling Behavior of Single-Sided Heated Concrete According to Compressive Strength and Heating Rate

**DOI:** 10.3390/ma14206023

**Published:** 2021-10-13

**Authors:** Euichul Hwang, Gyuyong Kim, Gyeongcheol Choe, Minho Yoon, Minjae Son, Dongkyun Suh, Hamin Eu, Jeongsoo Nam

**Affiliations:** 1 Department of Architectural Engineering, Chungnam National University, 99 Daehak-ro, Yuseong-gu, Daejeon 34134, Korea; sksdmlcjf@naver.com (E.H.); speed1382@gmail.com (G.C.); minjae931226@naver.com (M.S.); syhtw@naver.com (D.S.); wp05125@naver.com (H.E.); j.nam@cnu.ac.kr (J.N.); 2 R&D Project Management Department, Korea Agency for Infrastructure Technology Advancement, 286 Simin-daero, Dongan-gu, Anyang-si 14066, Korea; manho1201@kaia.re.kr

**Keywords:** heating rate, compressive strength, single-sided heat, vapor pressure, restrained stress, explosive spalling, ring-restrained concrete

## Abstract

In this study, the effects of heating rate and compressive strength on the spalling behavior of single-sided heated ring-restrained concrete with compressive strengths of 60 and 100 MPa were investigated. The vapor pressure and restrained stress inside the concrete were evaluated under fast- and slow-heating conditions. Regardless of the heating rate, the concrete vapor pressure and restrained stress increased as the temperature increased, and it was confirmed that spalling occurred in the 100-MPa concrete. Specifically, it was found that moisture migration and restrained stress inside the concrete varied depending on the heating rate. Under fast heating, moisture clogging and restrained stress occurred across the concrete surface, causing continuous surface spalling for the 100-MPa concrete. Under slow heating, moisture clogging occurred, and restrained stress continuously increased in the deep area of the concrete cross-section owing to the small internal temperature difference, resulting in explosive spalling for the 100-MPa concrete with a dense internal structure. Additionally, while the tensile strength of concrete is reduced by heating, stress in the heated surface direction is generated by restrained stress. The combination of stress in the heated concrete surface and the internal vapor pressure generates spalling. The experimental results confirm that heating rate has a significant influence on moisture migration and restrained stress occurrence inside concrete, which are important factors that determine the type of spalling.

## 1. Introduction

The internal structure of high-strength concrete is denser than that of normal-strength concrete. This increases the likelihood of spalling in high-strength concrete when it is exposed to high temperatures, such as temperatures caused by fire. Concrete spalling is difficult to predict owing to its irregular nature and may lead to strength degradation by causing cross-sectional loss of concrete members. It has been reported that vapor pressure, thermal stress, and a combination of the two are the main causes of concrete spalling [1,2,3,4,5].

In addition, the mechanical properties of concrete exposed to high temperatures are negatively affected. Concrete spalling and mechanical property degradation can have a significant effect on the structural stability of buildings. Various studies have been conducted to compensate for this phenomenon [6,7,8].

During heating, moisture migration occurs inside concrete, and the moisture is released outwards or condensed as it moves inwards. For high-strength concrete, the likelihood of spalling is high owing to low internal moisture migration and high vapor pressure on the surface [9,10,11]. Therefore, a method for vapor pressure reduction through moisture migration inside concrete has been proposed to inhibit spalling. In various studies, effective concrete vapor pressure reduction during heating has been experimentally confirmed for concrete fabricated by mixing polypropylene, nylon, polyethylene, jute fibers, and amorphous metallic fibers [11,12,13,14,15,16,17,18]. In addition, it has been reported that concrete mixed with steel fibers can suppress the explosion by increasing the tensile strength [19,20]. It is worth noting that these studies dealt with concrete spalling under fast-heating conditions.

Hertz [21] stated that the likelihood of spalling is generally increased by high temperatures and fast heating, but reported that spalling may occur in high-density concrete containing silica fume at a heating rate of 1 °C/min.

Algourdin et al. [22] heated concrete mixed with steel and polypropylene fibers at a rapid heating rate and at a rate of 10 °C/min. It was reported that the spalling was more severe in a cylindrical specimen than in a slab. Furthermore, in the absence of fibers, spalling may occur at a rate of 10 °C/min.

Phan et al. [23,24] applied a heating rate of 5 °C/min to concrete with w/cm values of 0.22, 0.33, and 0.57, and reported that the likelihood of spalling increased as the w/cm value decreased and that some high-strength concrete specimens exhibited spalling that destroyed them through an explosion. They defined such spalling as “explosive spalling” and spalling that involves continuous flaking from the concrete surface as “surface spalling.”

In addition, Choe et al. [25] examined the effect of heating rate on high-strength concrete spalling. They reported that the heating rate significantly affects moisture migration and vapor pressure accumulation in concrete. In particular, they found that moisture migration does not occur under slow-heating conditions owing to the similar temperature distribution in concrete. Thus, they reported that explosive spalling caused by boiling liquid expanding vapor explosion (BLEVE) may occur in high-strength concrete with a high-density matrix.

Recently, Ozawa et al. [26,27] devised an experimental method to estimate the vapor pressure and thermal stress in relation to the spalling mechanism. They restrained concrete with a ring-shaped steel pipe and estimated the thermal stress of concrete through the deformation of the ring-shaped steel pipe under single-side fast-heating. They reported that it was possible to evaluate the vapor pressure and restrained stress in concrete under fast-heating conditions using this experimental method.

**Figure 1:**
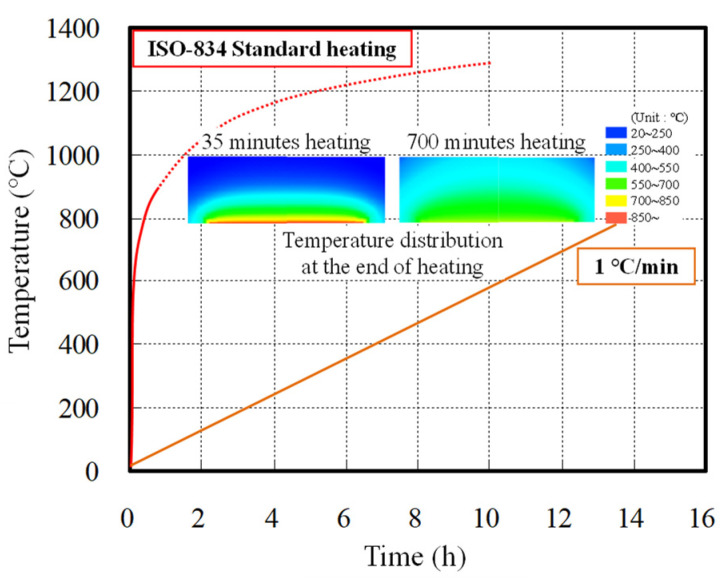
Temperature distribution of single-sided heated ring-restrained concrete according to heating rate.

Concrete spalling is significantly affected by the heating condition and compressive strength. In particular, to improve the fire safety performance of concrete structures, it is necessary to examine the concrete spalling behavior considering the heating condition and compressive strength. Previous studies focused on the spalling of concrete specimens by vapor pressure under heating; however, stress due to thermal expansion of concrete should be considered simultaneously. In addition, when concrete is subjected to single-sided heating, as shown in Figure 1, the internal temperature distribution changes and may affect the thermal properties of concrete. Therefore, it is necessary to investigate spalling by examining the vapor pressure and thermal stress in concrete subjected to single-sided heating and considering the heating rate and compressive strength.

In this study, ring-restrained concrete subjected to single-sided heating was used to investigate the concrete spalling behavior according to heating rate. When concrete with compressive strengths of 60 and 100 MPa were subjected to single-sided heating, the vapor pressure due to the temperature difference inside the concrete was measured, and the restrained stress by the ring-restrained condition was calculated to examine the influence of thermal stress.

## 2. Experiment

### 2.1. Experimental Plan

Table 1 shows the experimental plan followed in this study and Table 2 shows the mix proportions of concrete used in this study. To evaluate the vapor pressure and restrained stress in concrete according to heating rate, fast heating (ISO-834 standard heating method) and slow heating (RILEM TC 129-MHT “Part 3—Compressive strength for service and accident conditions” at a rate of 1 °C/min) were used [28,29]. In addition, for concrete with water-to-binder (W/B) ratios of 0.2 and 0.35, the spalling behavior, internal temperature, vapor pressure, and restrained stress were evaluated. The physical properties of the materials used for concrete are shown in Table 3.

**Figure 2:**
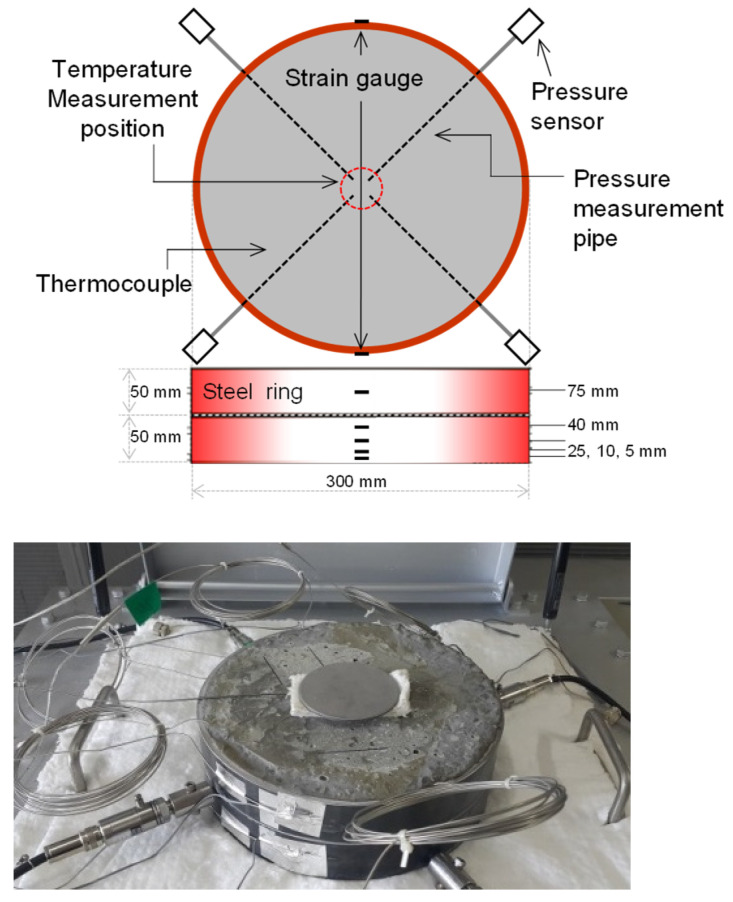
Overview of the ring-restrained concrete specimen.

Figure 2 shows an overview of the ring-restrained concrete specimen. A steel ring with a diameter, height, and thickness of 300, 50, and 7 mm, respectively, was used as a restraining ring. It was installed in two stages through bonding with silicone, and concrete was poured inside. In addition, thermocouples and pipes for pressure measurement were installed at depths of 5, 10, 25, and 40 mm from the heated surface of the concrete specimen. The deformation of the steel ring was measured by installing strain gauges for room temperature (80 ℃) at depths of 5, 10, 25, 40, and 75 mm from the heated surface of the concrete specimen. The fresh concrete specimens with compressive strengths of 60 and 100 MPa had slump flow values of 560 and 680 mm and an air content of 3.8 and 2.0%, respectively. Before heating, the concrete was cured for about 150 days to stabilize the moisture content, and the moisture content of the 60- and 100-MPa concrete specimens was stabilized at 3.95 and 4.20%. In addition, the ring-restrained concrete specimens were cured in a constant temperature and humidity chamber at a temperature of 22 ± 2 °C and humidity of 50% ± 10%.

### 2.2. Heating Method

**Figure 3:**
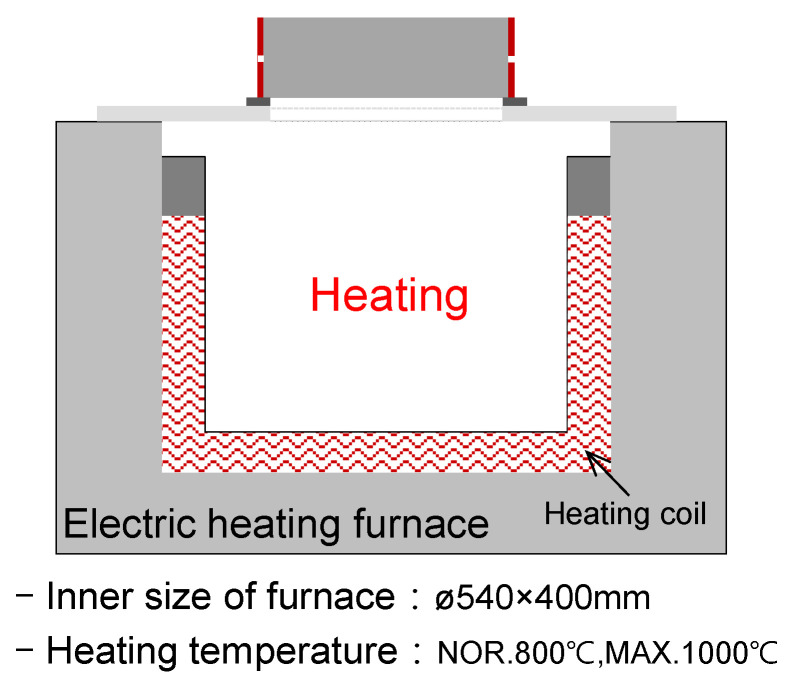
Electric heating furnace schematic and specifications.

Figure 3 shows a schematic of the electric heating furnace used in this study. A specimen was installed on top of the furnace, and single-sided heating was applied to the bottom of the specimen to perform fast and slow heating. The bottom of the ring was insulated to prevent the ring from being directly heated.

### 2.3. Calculation of Restrained Stress and Z-Axis Stress in Concrete

Restrained stress in concrete was calculated based on the research of Ozawa et al. [26,27], who estimated restrained stress based on thin-walled cylinder model theory [30]. This method involves measuring the deformation of the steel ring because concrete under heat-induced expansion causes the superficial deformation on the steel ring. Therefore, the restrained stress of the steel ring can be calculated as follows:

(1) $$\sigma_{re}=\varepsilon_{\theta }\times E_{s}\times \frac{t}{R}$$

where $\sigma_{re}$: restrained stress (MPa)

$\varepsilon_{\theta }$: circumferential strain of the steel ring

$E_{s}$: elastic modulus of the steel ring (MPa)

*t*, *R*: thickness and inside radius of the steel ring (mm)

**Figure 4:**
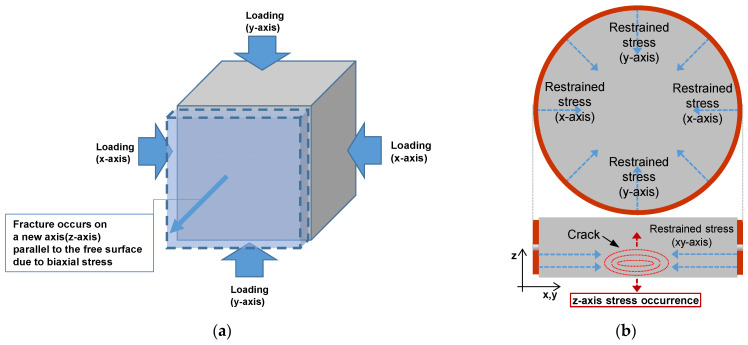
Restrained stress generated in concrete during heating. (**a**) New axis (*z*-axis) stress generation. (**b**) *z*-axis stress of concrete.

Figure 4 shows the restrained stress generated in ring-restrained concrete during heating. The *x*- and *y*-axis stress that corresponds to restrained stress occurs in concrete during heating, and the biaxial stress may generate stress in a new axis, as shown in Figure 4a. The *z*-axis stress, which occurs in the direction of the heated surface of the ring-restrained concrete specimens, may cause cracks inside the concrete, as shown in Figure 4b [26,27].

In concrete exposed to high temperatures, stress is generated by thermal expansion regardless of the axis. The *z*-axis stress in this study is a value estimated using the restrained stress and may be different from the actual stress in concrete; however, *z*-axis stress may occur and may affect the spalling. Therefore, *z*-axis stress can be calculated as follows:

(2) $$\sigma_{re}=\sigma_{x}=\sigma_{y}$$

(3) $$\tau_{xy}=0$$

(4) $$\sigma_{z}=v_{c}\left(\sigma_{x}+\sigma_{y}\right)$$

where $\sigma_{x},\sigma_{y}$: stress normal in the orthogonal *x*-*y* coordinate (MPa)

$\tau_{xy}$: shear stresses in the orthogonal *x*-*y* coordinate

$v_{c}$: apparent Poisson’s ratio of concrete

$\sigma_{z}$: stress at a certain depth from the heated surface (MPa)

## 3. Experimental Results and Discussion

### 3.1. Spalling Type and Internal Temperature

**Figure 5:**
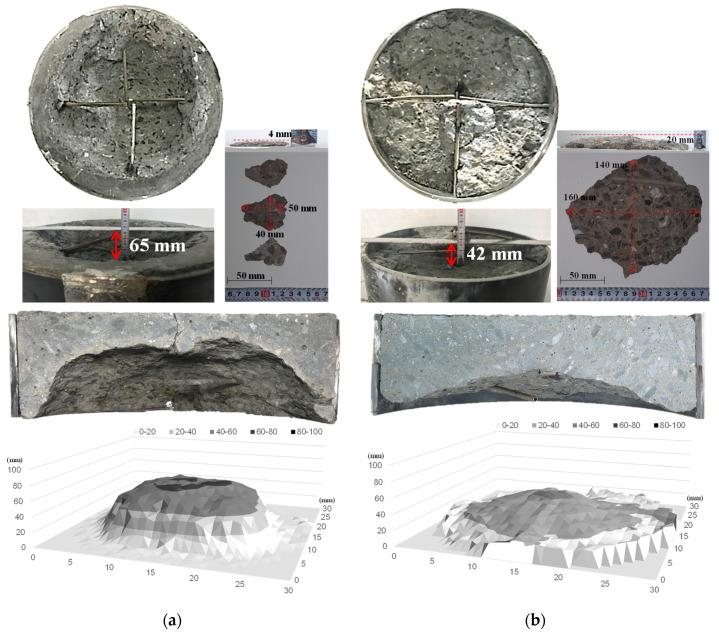
Spalling behavior of 100-MPa concrete according to heating rate. (**a**) Fast heating (ISO-824). (**b**) Slow heating (1 °C/min).

Figure 5 shows the spalling behavior of the 100-MPa concrete specimen according to heating rate. Spalling occurred in the 100-MPa concrete specimen in the fast- and slow-heating experiments. As shown in Figure 5a, 100-MPa concrete exhibited surface spalling in which small debris separated from the center of the heated surface under fast heating. Continuous surface spalling occurred, resulting in a maximum spalling depth of approximately 65 mm.

However, under slow heating, the 100-MPa concrete specimen exhibited explosive spalling that caused a large internal fracture, resulting in a maximum spalling depth of approximately 42 mm. In addition, the maximum debris size differed depending on the spalling type. The debris size was approximately 50 × 40 × 4 mm for surface spalling and 160 × 140 × 20 mm for explosive spalling, showing that explosive spalling had a larger debris size.

For the spalling depth distribution of the 100-MPa concrete specimen for different spalling types, the spalling depth was measured in the form of a grid on the heated surface of the concrete specimen. Under fast heating, concrete was peeled off from the center of the heated surface owing to surface spalling, but the concrete close to the steel ring exhibited a narrow and deep shape without spalling. This appears to be because spalling occurred in the center of the concrete specimen where the temperature rapidly increased, and thus the expansion force of the concrete close to the steel ring was relieved. Under slow heating, explosive spalling temporarily occurred, but a wide and shallow shape was observed compared to surface spalling. In addition, the weight loss of the concrete was found to be approximately 25.40% and 21.25% for surface and explosive spalling, respectively. It was confirmed that explosive spalling can exhibit a similar amount of weight loss to that of surface spalling, which occurs continuously despite being a one-time phenomenon.

**Figure 6:**
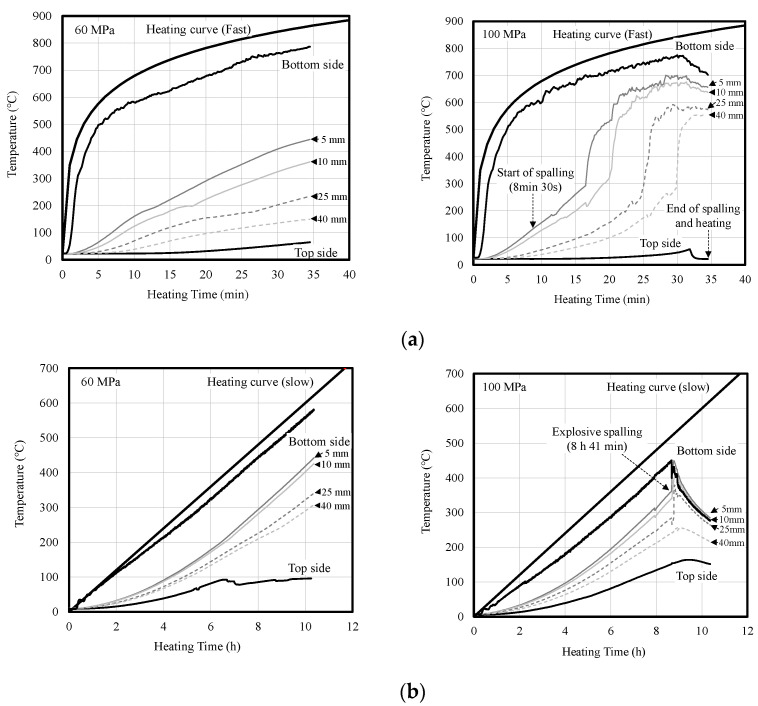
Internal temperature of concrete according to heating rate. (**a**) Fast heating (ISO-824). (**b**) Slow heating (1 °C/min).

Figure 6 shows the internal temperature of concrete according to heating rate. Under fast heating, the temperature difference inside the concrete was large regardless of the compressive strength of the concrete. For the 60-MPa concrete specimen that did not exhibit spalling, a temperature difference of approximately 300 °C was observed between the depths of 5 and 40 mm at the end of heating. In addition, the 100-MPa concrete specimen exhibited surface spalling at approximately 8 min and 30 s after the start of heating, and the thermocouple at a depth of 5 mm showed a sharp temperature change at approximately 17 min. It appears that this thermocouple was exposed by surface spalling. The thermocouples were exposed at temperatures approaching 300 °C by continuous surface spalling in the order of 10, 25, and 40 mm depths.

Under slow heating, the temperature difference inside the concrete was smaller than that under fast heating. For the 60-MPa concrete specimen that did not exhibit spalling, a temperature difference of approximately 130 °C was observed between the depths of 5 and 40 mm at the end of heating. In addition, the 100-MPa concrete specimen exhibited explosive spalling at approximately 8 h and 41 min after the start of heating. Because of this spalling, the thermocouples at the depths of 5, 10, and 25 mm were exposed simultaneously, thereby exhibiting sharp temperature changes.

### 3.2. Vapor Pressure

**Figure 7:**
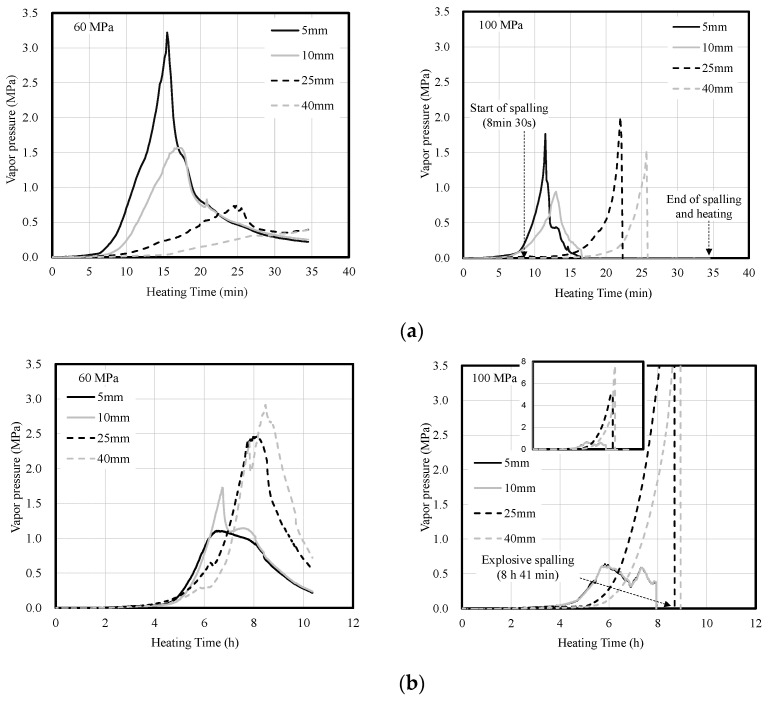
Vapor pressure of concrete according to heating rate. (**a**) Fast heating (ISO-824). (**b**) Slow heating (1 °C/min).

Figure 7 shows the vapor pressure of concrete according to heating rate. Under fast heating, the vapor pressure inside the concrete rapidly increased and then decreased in the order of 5, 10, 25, and 40 mm depths regardless of the compressive strength of the concrete. This appears to be due to the large temperature difference inside the concrete caused by fast heating. In addition, it appears that the vapor pressure was released without causing spalling owing to internal cracks for the 60-MPa concrete specimen, whereas the vapor pressure was discharged owing to surface spalling for the 100-MPa concrete specimen.

Under slow heating, the vapor pressure inside the concrete slowly increased. In particular, for the 100-MPa concrete specimen, the vapor pressure at the depths of 5 and 10 mm was released, but the vapor pressure at 40 mm increased to 7.6 MPa. The vapor pressure decreased after explosive spalling.

**Figure 8:**
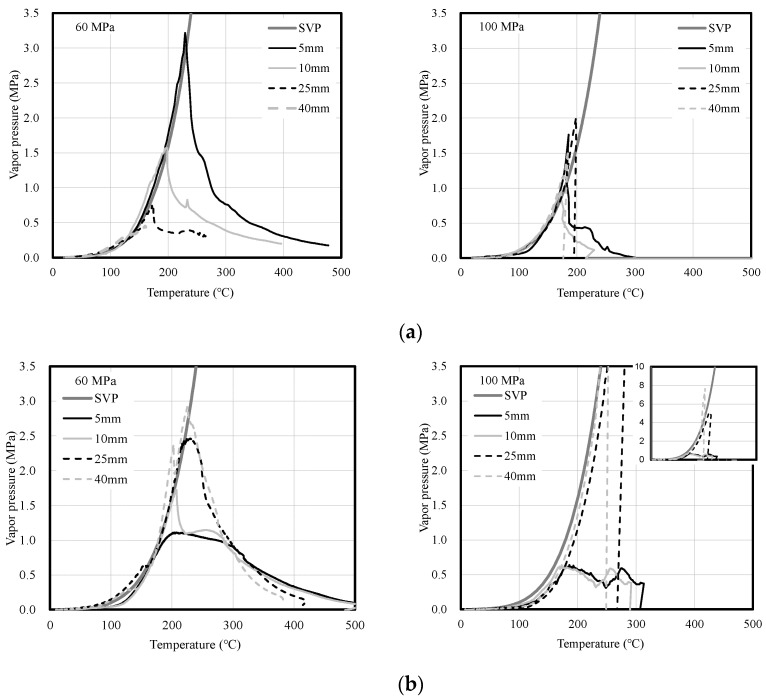
Comparison of the vapor pressure of concrete and the SVP curve according to heating rate. (**a**) Fast heating (ISO-824). (**b**) Slow heating (1 °C/min).

Meanwhile, as shown in Figure 8, the vapor pressure and the saturated water vapor pressure (SVP) curve were compared to examine moisture migration inside the 60- and 100-MPa concrete specimens according to heating rate. SVP represents the maximum vapor pressure at a specific temperature, and the vapor condition can be divided into three states through comparison. The first is the supersaturated state, in which the vapor pressure is higher than the saturated vapor pressure. Owing to the inflow of moisture from the outside, more vapor exists than the amount that can be contained at the measurement location. The second is the state in which the vapor pressure is equal to the saturated vapor pressure. No vapor is introduced from the outside, and moisture does not move from the measurement location to a different location. The third is the unsaturated state, in which the vapor pressure is lower than the saturated vapor pressure. In this case, the amount of vapor is insufficient. Therefore, it is possible to examine the moisture state change, amount of vapor, and moisture migration in concrete during heating by comparing the saturated vapor pressure of concrete with its vapor pressure [31].

Under fast heating, the vapor pressure was similar to the SVP curve or exhibited a supersaturated state regardless of the compressive strength of the concrete. Owing to the fast heating, the vapor generated from the heated surface is discharged outwards or moves inwards, and the moisture that moves inwards forms moisture clogging by causing the supersaturated state [32,33]. Because the 100-MPa concrete specimen has a denser internal structure than the 60-MPa one, moisture clogging is formed from the heated surface of the concrete. Furthermore, for the 100-MPa concrete specimen, moisture clogging is formed by supersaturated vapor at depths between 5 and 40 mm, thereby causing continuous surface spalling.

Under slow heating, the 60-MPa concrete specimen exhibited a saturation state similar to the SVP curve, whereas the 100-MPa concrete specimen showed the unsaturated state except for the vapor pressure at 40 mm. The vapor pressure slowly increased under slow heating regardless of the compressive strength of concrete. This delayed the time of the vapor pressure release, and moisture moved to a deep location away from the heated surface of the concrete specimen. The migrated moisture formed moisture clogging by causing a supersaturated state at a depth of 40 mm in the 100-MPa concrete specimen, and the vapor pressure was more than twice as high as that under fast heating. Explosive spalling occurred owing to the vapor condensing inside the concrete.

### 3.3. Restrained Stress in Ring-Restrained Concrete

**Figure 9:**
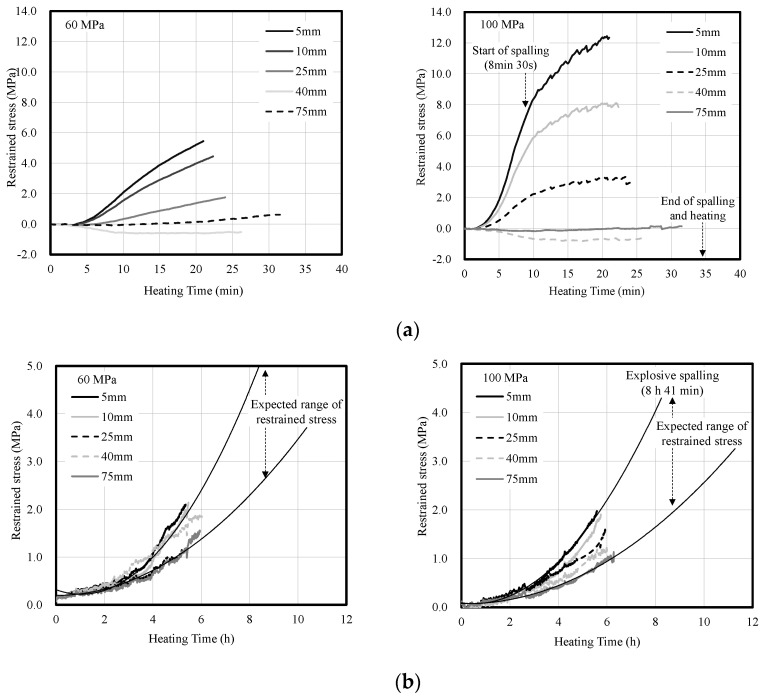
Restrained stress in concrete according to heating rate. (**a**) Fast heating (ISO-824). (**b**) Slow heating (1 °C/min).

Figure 9 shows the restrained stress in the concrete according to heating rate. Under fast heating, the restrained stress in the concrete increased with the heating temperature regardless of the compressive strength of the concrete, but the maximum restrained stress of the 100-MPa concrete specimen was significantly higher than that of the 60-MPa concrete specimen. Owing to the temperature difference inside the concrete, the restrained stress was higher toward the heated surface. In addition, shrinkage deformation occurred at the 40-mm depth. It appears that the shrinkage deformation was caused by thermal expansion that occurred at depths of 5 to 25 mm.

Under slow heating, restrained stress tended to slowly increase regardless of the compressive strength of the concrete because the temperature difference inside the concrete was small compared to that under fast heating. As the strain gauges of the steel ring could only be used for temperatures of up to approximately 80 °C, the expansion deformation could only be measured for approximately 6 h. However, it can be estimated that the restrained stress continued to increase afterwards.

For the 100-MPa concrete specimen that exhibited spalling, stress in the direction of the heated surface (*z*-axis stress) may occur as the restrained stress of the heated surface increases, as explained in Section 2.3. The *z*-axis stress may cause cracks inside the concrete, and it acts as a force that pushes the concrete surface in the direction of the heated surface. Spalling occurs owing to a combination of the *z*-axis stress and vapor pressure, and it appears that the influence of the *z*-axis stress is different depending on the spalling type.

### 3.4. Spalling Behavior According to Vapor Pressure and Restrained Stress

**Figure 10:**
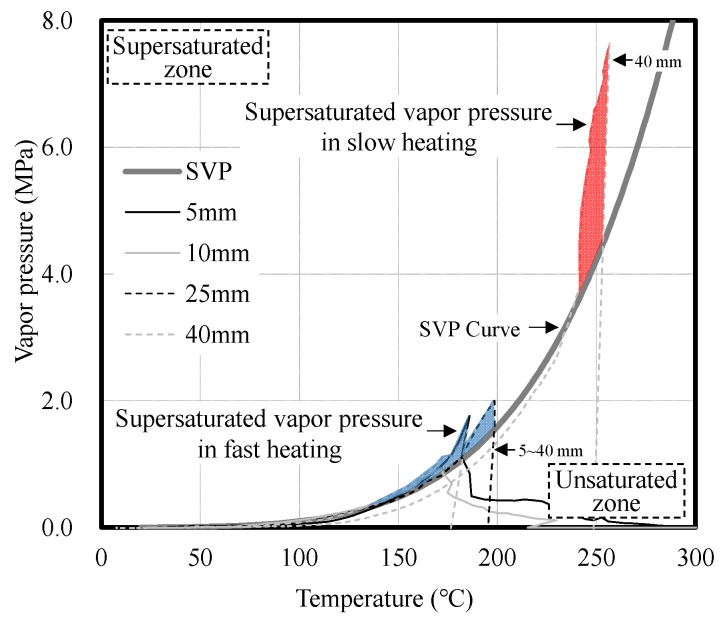
Vapor pressure in the 100-MPa concrete specimen that exhibited spalling.

Figure 10 shows the vapor pressure in the 100-MPa concrete specimen that exhibited spalling. Under fast heating, supersaturated vapor was generated at depths of 5 to 40 mm. Moisture clogging was formed by supersaturated vapor from approximately 130 °C at all locations, and a maximum of 2.0 MPa was observed. Under slow heating, moisture clogging of approximately 7.6 MPa was formed by supersaturated vapor at a depth of 40 mm. Moisture clogging that increased from approximately 230 °C showed a significant increase compared to that under fast heating. Compared to fast heating, the temperature of the concrete increased very slowly under slow heating. Thus, the moisture inside the concrete moved slowly and finally formed moisture clogging at a depth of 40 mm. The moisture clogging formed inside the concrete pushes the concrete in the direction of the heated surface and acts as the main cause of spalling.

**Figure 11:**
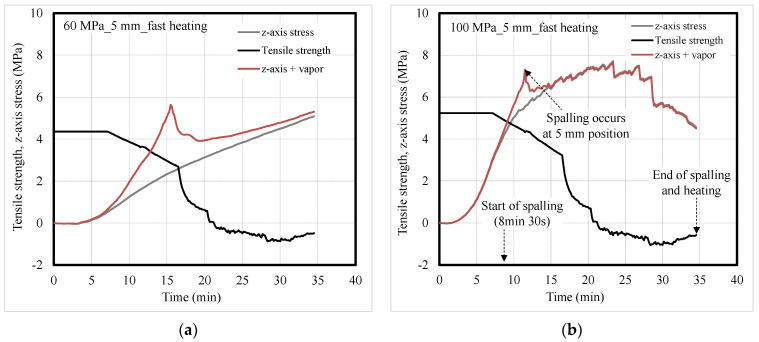
Tensile strength and *z*-axis stress of concrete under fast heating; (**a**) 60-MPa and (**b**) 100-MPa specimens.

**Figure 12:**
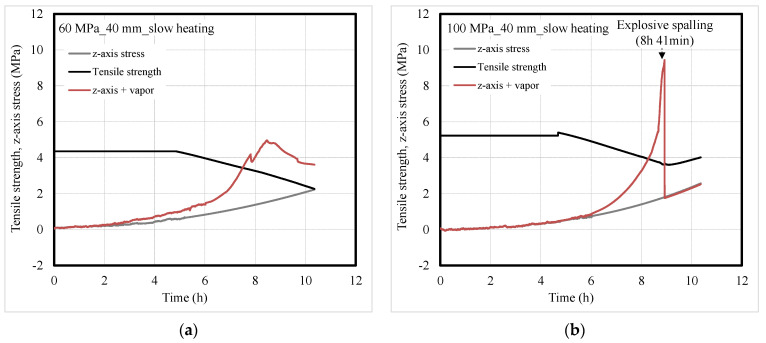
Tensile strength and *z*-axis stress of concrete under slow heating; (**a**) 60-MPa and (**b**) 100-MPa specimens.

Figure 11 and Figure 12 show the tensile strength and the *z*-axis stress of concrete according to heating rate. The values at a depth of 5 mm were compared for fast heating that caused surface spalling, while those at a depth of 40 mm were compared for slow heating that led to explosive spalling. Eurocode was used for the residual tensile strength of concrete exposed to high temperature [34], and Poisson’s ratio was set to 0.3 for the concrete during heating [35]. As the heating temperature increased, the tensile strength of the concrete decreased while the *z*-axis stress in the concrete increased [36].

Under fast heating, *z*-axis stress in the concrete rapidly increased and was higher for the 100-MPa concrete specimen than for the 60-MPa one. As the heating temperature increased, the *z*-axis stress and vapor pressure in the concrete increased, and they exceeded the residual tensile strength of concrete. This phenomenon occurred earlier in the 100-MPa concrete specimen that exhibited spalling than in the 60-MPa concrete specimen that did not exhibit spalling. For the 100-MPa concrete specimen, surface spalling began at approximately 8 min 30 s. Then, the sum of the *z*-axis stress and vapor pressure at a depth of 5 mm significantly exceeded the residual tensile strength of concrete, thereby causing surface spalling. It appears that the 100-MPa concrete specimen exhibited continuous surface spalling by repeating the above phenomenon. For the 60-MPa concrete specimen, spalling did not occur because the vapor was discharged without being expanded to the supersaturated state, even though the sum of the *z*-axis stress and vapor pressure exceeded the residual tensile strength of the concrete.

Under slow heating, the *z*-axis stresses in the concrete were found to be similar regardless of the compressive strength. As the heating temperature increased, the *z*-axis stress and vapor pressure in the concrete increased and exceeded the residual tensile strength of the concrete. This phenomenon occurred earlier in the 60-MPa concrete specimen than in the 100-MPa one. However, the 60-MPa concrete specimen did not exhibit spalling because the vapor was released before being expanded to the supersaturated state as in fast heating. For the 100-MPa concrete specimen, the residual tensile strength of the concrete was significantly exceeded owing to a rapid increase in the supersaturated vapor at a depth of 40 mm, resulting in explosive spalling at approximately 8 h 41 min.

**Figure 13:**
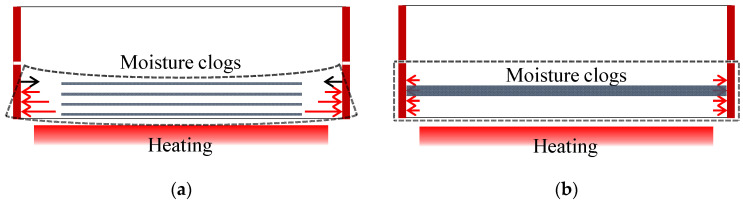
Schematic of vapor pressure and restrained stress of 100-MPa concrete according to heating rate. (**a**) Fast heating (ISO-824). (**b**) Slow heating (1 °C/min).

As shown in Figure 13, the heating rate affects the moisture migration and restrained stress in concrete. For the 100-MPa concrete specimen with a dense internal structure, moisture migration is not active and the supersaturated vapor inside forms moisture clogging. In addition, the continuous increase in heat-induced restrained stress generates *z*-axis stress. It was confirmed that the supersaturated vapor and restrained stress in concrete are the main factors of spalling, and that surface or explosive spalling may occur in concrete depending on the heating rate and compressive strength.

## 4. Conclusions

In this study, single-sided heating was applied to ring-restrained concrete to evaluate its vapor pressure and restrained stress, which are the main causes of spalling. Spalling was significantly affected by the compressive strength and heating rate of the concrete. Based on our experimental results, the following conclusions were drawn.

1. Because concrete has a low thermal conductivity, an internal temperature difference occurs depending on the heating rate. In particular, the temperature gradient of concrete may increase under a single-sided heating condition. The temperature difference inside concrete affects the formation of vapor pressure and restrained stress, which are important factors that determine the spalling type. The temperature difference inside concrete is large and continuous surface spalling occurs under fast heating, whereas the temperature difference inside concrete is small and explosive spalling occurs under slow heating.
2. Under fast heating, the heated surface of concrete is continuously peeled off and small concrete debris is generated in large quantities owing to the surface spalling of concrete. However, under slow heating, explosive spalling of concrete involves an impact and generates a very large concrete fracture. In particular, explosive spalling may cause rapid strength degradation because it may lead to a rapid cross-sectional loss. It was confirmed that the spalling type of concrete has a significant influence on the cross-sectional loss pattern.
3. For high-strength concrete with a dense internal structure, moisture migration occurs when the internal temperature increases, and the inward-migrated moisture forms moisture clogging in the supersaturated state. Under fast heating, the temperature difference in concrete between the surface and the inside was large, therefore, repeated surface spalling occurred owing to the moisture clogging formed on the heated surface of the concrete and the rapidly increasing restrained stress. Under slow heating, moisture migration was not active because the temperature was evenly distributed from the surface to the inside of the concrete, but explosive spalling occurred owing to the moisture clogging forming in a deep area of the concrete and the restrained stress slowly increased. The moisture clogging and restrained stress formed by the temperature distribution in high-strength concrete significantly affect the spalling type.
4. Concrete exhibited surface or explosive spalling depending on the heating condition and compressive strength. The cross-sectional loss pattern varies depending on the spalling type and can affect the strength degradation of concrete. For surface spalling, the strength of concrete can be conserved if part of the cross-sectional loss is inhibited. However, the occurrence of explosive spalling may cause a rapid strength degradation of concrete. As the strength degradation of concrete affects the structural stability of concrete structures, it is necessary to control concrete spalling considering the heating condition and compressive strength.

In future work, spalling control methods according to heating rate should be investigated. In addition to previous methods, new methods and materials for explosion control should be considered. Furthermore, there is also a need to study the effectiveness of concrete members.

## Figures and Tables

**Table 1:** Experimental plan for this study.

f_ck_ (MPa)	Heating Method	Test Item
60 100	Fast Heating (ISO-834) Slow Heating (1 °C/min.)	Spalling behavior Temperature (°C) Vapor pressure (MPa) Restrained stress (MPa)

**Table 2:** Mix proportion of concrete used in this study.

W/B	f_ck_ (MPa)	Slump Flow (mm)	Air (%)	S/a (%)	Unit Weight ^(^^1)^ (kg/m^3^)					
					W	C	FA	SF	S	G
0.35	60	650 ± 100	4	40	165	471	0	0	681	1026
0.20	100	750 ± 100	2	43	150	525	150	75	642	870

^(1)^ W: Water, C: Cement, FA: Fly ash, SF: Silica fume, S: Fine aggregate, G: Coarse aggregate.

**Table 3:** Physical properties of the materials used in this study.

Material	Physical Property
Cement	OPC (density: 3.15 g/cm^3^, specific surface area: 3200 cm^2^/g)
Fly ash	Density: 2.20 g/cm^3^, specific surface area: 3000 cm^2^/g
Silica fume	Density: 2.50 g/cm^3^, specific surface area: 200,000 cm^2^/g
Fine aggregate	Sea sand (density: 2.65 g/cm^3^, absorption: 1.00%)
Coarse aggregate	Crushed granitic aggregate (size: 20 mm, density: 2.62 g/cm^3^, absorption: 0.8%)
Super plasticizer	Polycarboxylic-based super plasticizer
